# JMCC plus - Celebrating milestones and looking ahead in our third year

**DOI:** 10.1016/j.jmccpl.2025.100286

**Published:** 2025-01-29

**Authors:** Davor Pavlovic, Rebekah Gundry

**Affiliations:** a Department of Cardiovascular Sciences, University of Birmingham, United Kingdom; b University of Nebraska Medical Center, Omaha, NE, United States of America

As we enter the third year of publishing the *Journal of Molecular and Cellular Cardiology Plus (JMCC Plus)*, we are thrilled to share some significant milestones that mark a new phase in our journey. These achievements solidify the journal's position as an important platform for advancing cardiovascular science, and are a testament to the collective efforts of our contributors, reviewers, editors, administrators and the leadership of the International Society for Heart Research (ISHR).

**Inclusion in the Web of Science** - One of our most notable accomplishments is the selection of JMCC Plus for inclusion in the Web of Science. This recognition means that we will receive our first Impact Factor in June 2025 - a pivotal achievement that reflects the growing influence and visibility of *JMCC Plus* in the scientific community.

**Indexing in PubMed Central (PMC)** - We are also delighted to announce that JMCC Plus has been accepted for indexing in PMC. This ensures that all manuscripts published in the journal are automatically listed on PubMed, significantly increasing their accessibility and reach to researchers worldwide.

**Acceptance for Indexing by Scopus** - Additionally, JMCC Plus has been accepted for indexing by Scopus, further enhancing our global visibility and credibility. With this achievement, we anticipate receiving our first CiteScore in June 2025, providing another metric of the journal's impact.

**Key Highlights from Our Journey** - Since our inception, JMCC Plus has published 11 volumes, showcasing innovative research, reviews and commentary that span molecular, cellular, and translational cardiovascular physiology/pathophysiology. These volumes represent the dedication and expertise of a vibrant and engaged research community.

Another milestone in our efforts to foster dialogue and innovation was the successful hosting of our first webinar, titled “Use and Abuse of AI and AI Language Models in Cardiovascular Research.” This event, available for viewing on our YouTube channel (link here), brought together thought leaders to discuss a critical and timely topic, further cementing JMCC Plus as a forum for addressing emerging challenges and opportunities in the field.

**Conference Abstract Publications** - We are proud to have published the abstracts from key ISHR meetings in 2024, including:

• The ISHR European Section meeting held in beautiful Toulouse in June 2024 (View Abstracts).
• The newly formed South East Asia Section meeting held in stunning Singapore in October 2024 (View Abstracts).
• Soon, we will publish the abstracts from the North American Section meeting held in August 2024 in trendy Long Beach, California.

**Special Issues** - We have launched three new special issues:

• Omics in Heart, Vessel and Brain Diseases, guest edited by Prof. Yvan Y. Devaux.
• Unveiling the Potential of Comparative Physiology to Treat Cardiac Diseases, guest edited by Dr. Julien Ochala.
• 2024 ISHR-NAS Section Meeting Special Issue, guest edited by Dr. Erik Blackwood and Dr. Timothy O'Connell.

Details about these special issues can be found here.

We encourage researchers interested in proposing additional special issues to contact our editorial team at d.pavlovic@bham.ac.uk or rebekah.gundry@unmc.edu. We look forward to collaborating on innovative topics that advance the field of cardiovascular research.

**Reproducibility and Reporting of Negative Results in Cardiovascular Research** - At JMCC Plus, we believe that reproducibility and the reporting of negative results are essential components of advancing research. As part of our ethos, we are committed to providing a platform that values transparency and rigour in scientific enquiry and strongly believe that publishing reproducible research and well-documented negative findings not only helps prevent redundancy but also guides the scientific community toward more targeted and impactful investigations. By embracing these principles, we aim to foster a culture of honesty and progress that benefits the society as a whole. With that in mind we extend our call for papers reporting on reproducibility and negative findings, please submit your manuscripts relevant for this call using our submission portal.

**Social Media Engagement** - In our efforts to enhance engagement and visibility, JMCC Plus is active on multiple social media platforms. You can follow us on X (formerly Twitter), our recently launched profile on Bluesky (View Profile) and WeChat in China. We are also excited to announce that we will soon be joining LinkedIn to further connect with the global cardiovascular research community. For this we thank our hard working social media editors, Dr. Daniel Johnson, Dr. Yihua Bei and Ms. Olivia Baines.

**Open Access Fee Waiver Extension** - We are pleased to announce that the open access fee waiver for articles submitted before March 31, 2025, has been extended. Extension underscores our commitment to making high-quality cardiovascular research widely accessible. After this date, articles submitted between April 1, 2025, and September 30, 2025, will receive a 50 % discount on the APC and from September 30, 2025, a standard open access fee of $2020 will apply.

**Gratitude and Vision for the Future** - These achievements would not have been possible without the unwavering support and trust of our authors, reviewers, editors, administrators, and the ISHR leadership. We extend our deepest gratitude to everyone who has contributed to the journal's success.

Looking ahead, we remain committed to providing a platform for groundbreaking cardiovascular research and fostering collaboration across the global scientific community. With our inclusion in major indexing platforms and ongoing efforts to promote high-quality science, the future of JMCC Plus is brighter than ever.

Thank you for being part of our journey. We look forward to another year of progress, discovery, and impact.

## CRediT authorship contribution statement

**Davor Pavlovic:** Writing – original draft. **Rebekah Gundry:** Writing – review & editing.

